# Supplementary material for: Slow magnetic relaxation in a europium(II) complex
Source: Nat Commun. 2024 Apr 8;15:3010. doi: 10.1038/s41467-024-46196-w (PMC11001981; doi:10.1038/s41467-024-46196-w)
Supplement: Supplementary file 1 — Supplementary Information [file 41467_2024_46196_MOESM1_ESM.docx]

Supplementary Information

**Slow Magnetic Relaxation in a Europium(II) Complex**

Dylan Errulat^†1^, Katie L. M. Harriman^†1^, Diogo A. Gálico^1^, Elvin V. Salerno^2^, Johan van Tol^2^, Akseli Mansikkamäki^3^, Mathieu Rouzières^4^, Stephen Hill*^2,5^, Rodolphe Clérac*^4^, and Muralee Murugesu*^1^

*^1^* Department of Chemistry and Biomolecular Sciences, University of Ottawa, Ottawa, Ontario K1N 6N5, Canada.

*^2^* National High Magnetic Field Laboratory, Florida State University, Tallahassee, FL 32310, USA

*^3^* NMR Research Unit, University of Oulu, P. O. Box 3000, 90014 Oulu, Finland.

*^4^* Univ. Bordeaux, CNRS, CRPP, UMR 5031, F-33600 Pessac, France.

*^5^* Department of Physics, Florida State University, Tallahassee, FL 32306, USA.

Corresponding Authors: Muralee Murugesu* (E-mail: m.murugesu@uottawa.ca), Rodolphe Clérac* (E-mail: rodolphe.clerac@u-bordeaux.fr) & Stephen Hill* (E-mail: shill@magnet.fsu.edu)

^†^ These authors contributed equally to this work.

**Table of Contents**

Additional Crystallography Figures and Tables...........................................................................	S3

Figures and Details of the Luminescence Measurements............................................................	S5

Additional Figures and Tables of the Magnetic Measurements...................................................	S7

References...................................................................................................................................	S14

**Additional Crystallography Figures and Tables**

**Supplementary Table 1.** Crystallographic Data for Eu{N(SiMePh_2_)_2_}_2_ (**1**).

|  | **Eu{N(SiMePh_2_)_2_}_2_**  (**1**) |
| --- | --- |
| Empirical formula | C_52_H_52_EuN_2_Si_4_ |
| Formula weight | 969.27 |
| Temperature /K | 200.05 |
| Crystal system | Monoclinic |
| Space group | *P*2_1_/c |
| *a* /Å | 10.9266(5) |
| *b* /Å | 15.3101(6) |
| *c* /Å | 27.9764(12) |
| *α* /^o^ | 90 |
| *β* /^o^ | 93.625(1) |
| *γ* /^o^ | 90 |
| Volume /Å^3^ | 4670.7(3) |
| *Z* | 4 |
| *ρ*_calc_ g/cm^3^ | 1.378 |
| *µ* /mm^-1^ | 1.482 |
| *F*(000) | 1988.0 |
| Crystal size /mm^3^ | 0.137 × 0.13 × 0.127 |
| Radiation | MoKα (λ = 0.71073) |
| 2*θ* range for data collection /^o^ | 2.918 to 56.69 |
| Index ranges | -14 ≤ *h* ≤ 14  -20 ≤ *k* ≤ 20  -37 ≤ *l* ≤ 37 |
| Reflections collected | 73413 |
| Independent reflections | 11648 [*R*_int_ = 0.0662, *R*_sigma_ = 0.0502] |
| Data/restraints/parameters | 11648/0/536 |
| Goodness-of-fit on *F*^2^ | 1.004 |
| Final *R* indexes [*I* ≥ 2*σ*(*I*)] | *R*_1_ = 0.0421, *wR*_2_ = 0.0755 |
| Final *R* indexes [all data] | *R*_1_ = 0.0863, *wR*_2_ = 0.0909 |
| Largest diff. peak/hole / e Å^-3^ | 1.60/-1.55 |

**Supplementary Figure 1.** Unit cell packing diagrams along the a-, b- and c-axis of Eu{N(SiMePh2)2}2 (**1**).

**Supplementary Figure 2.** Excitation spectrum for Eu{N(SiMePh2)2}2 (**1**) obtained at 10 K (*λ*em = 518 nm).

**Supplementary Figure 3.** (a) Emission spectra for **1** obtained at 10 and 293 K (*λ*_ex_ = 270 nm). (b) Deconvolution of the 293 K emission spectrum.

**Supplementary Figure 4.** Room-temperature emission decay (black points) and mono exponential function fit (red line) for Eu{N(SiMePh2)2}2 (**1**).

**Supplementary Figure 5.** Emission spectra for the crude oxidized product of Eu{N(SiMePh2)2}2 (**1**) obtained in C6H5F (*λ*ex = 270 nm).

**Additional Figures and Tables of Magnetic Measurements**

**Supplementary Figure 6.** Linear (left) and semi-logarithm (right) *χT* versus *T* plots at 1 kOe and 10 kOe between 1.85 and 300 K for Eu{N(SiMePh2)2}2 (**1**) (*χ* is defined as the magnetic susceptibility equal to *M/H* per mole of complex). At 1 kOe, the 300 K and 1.85 K *χT* products are respectively 7.8 and 6.3 cm^3^ K/mol for Eu{N(SiMePh2)2}2 (**1**).

**Supplementary Figure 7.** Field dependence of the magnetization shown as *M* versus *H* (left) and *M* versus *H/T* (right) plots for Eu{N(SiMePh2)2}2 (**1**) below 8 K.

**Supplementary Figure 8.** Attempted fit of the experimental *χT* versus *T* (left) and *M* versus *H* (right) data to a *S* = 7/2 approximation with the following spin Hamiltonian: $H=DS_{T,z}^{2}$, calculated with the software PHI.**^1^**

**Supplementary Figure 9.** Frequency dependence of the in-phase (*χ*'; left) and out-of-phase (*χ*''; right) components of the ac susceptibility for Eu{N(SiMePh2)2}2 (**1**) between 0 and 10 kOe for ac frequencies between 10 Hz and 10 kHz at 2 K. Solid lines represent the fits to the generalized Debye model.

**Supplementary Figure 10.** Field dependence of the fitting parameters *α*, *ν*, *χ*_0_, *χ*_∞_, and *χ*_0_-*χ*_∞_, between 0 and 5 kOe at 2 K determined from the generalized Debye fit of the frequency dependence of the in-phase (*χ*') and out-of-phase (*χ*'') components of the ac susceptibility (Supplementary Figure 9).

**Supplementary Figure 11.** Field dependence of the average relaxation times (*τ*) between 0 and 4 kOe at 2 K determined from the generalized Debye fit of the frequency dependence of the in-phase (*χ*') and out-of-phase (*χ*'') components of the ac susceptibility (Supplementary Figure 9). The red line represents the best-fit to QTM relaxation, with parameters *B*_1_ = 1.8(1) 10^4^ s^-1^, *B*_2_ = 22.8(2.3) kOe^-2^ (Equations 1 & 2 without Raman term, and with the Orbach term set as a constant).

**Supplementary Figure 12.** Temperature dependence of the in-phase (*χ*'; left) and out-of-phase (*χ*''; right) components of the ac susceptibility for Eu{N(SiMePh2)2}2 (**1**) between 10 Hz and 10 kHz for the temperature range 1.9-15 K under an applied static field of 800 Oe.

**Supplementary Figure 13.** Frequency dependence of the in-phase (*χ*'; left) and out-of-phase (*χ*''; right) components of the ac susceptibility for Eu{N(SiMePh2)2}2 (**1**) between 1.9 and 8 K for ac frequencies between 10 Hz and 10 kHz under an applied static field of 800 Oe. Solid lines represent the fits to the generalized Debye model.

**Supplementary Figure 14.** Temperature dependence of the fitting parameters *α*, *ν*, *χ*_0_, *χ*_∞_, and *χ*_0_-*χ*_∞_, between 1.9 and 8 K under an applied static field of 800 Oe determined from the generalized Debye fits of the frequency dependence of the in-phase (*χ*') and out-of-phase (*χ*'') components of the ac susceptibility (Supplementary Figure 13).

**Supplementary Figure 15.** Temperature dependence of -ln(*τ*) between 1.9 K – 5 K and 800 Oe determined from the generalized Debye fit of the frequency dependence of the in-phase (*χ*') and out-of-phase (*χ*'') components of the ac susceptibility (Supplementary Figure 13). The red line represents the best-fit to relaxation by a combination of Orbach and QTM relaxation (See main text; Equation 1 & 2 without Raman and direct terms). The grey vertical solid bars on the plot represent the ESD of the relaxation time and have been calculated from the parameters of the generalized Debye fit and the log-normal distribution as described in Reference 2.

**Supplementary Equation 1.**

$\chi'(\upsilon) ={\chi'}_{\infty} + ({\chi'}_{0} - {\chi'}_{\infty}) \frac{1 + {(\omega\tau)}^{1-\alpha} sin(\alpha\pi/2)}{1 + {2\left( 2\pi\upsilon\tau\right)}^{1-\alpha} sin\left( \frac{\alpha\pi}{2} \right) +\left( 2\pi\upsilon\tau\right)^{2(1-\alpha)}}$

**Supplementary Equation 2.**

$\chi''(\upsilon) =({\chi'}_{0} - {\chi'}_{\infty}) \frac{{(2\pi\upsilon\tau)}^{1-\alpha} cos(\alpha\pi/2)}{1 + {2\left( 2\pi\upsilon\tau\right)}^{1-\alpha} sin\left( \frac{\alpha\pi}{2} \right) +\left( 2\pi\upsilon\tau\right)^{2(1-\alpha)}}$

**Supplementary References**

[1] Chilton, N.F et al. PHI: a powerful new program for the analysis of anisotropic monomeric and exchange-coupled polynuclear d- and f-block complexes. *J. Comput. Chem.* **34**, 1164–1175 (2013).

[2] Reta, D. & Chilton, N. F. Uncertainty estimates for magnetic relaxation times and magnetic relaxation parameters. *Phys. Chem. Chem. Phys.* **21**, 23567–23575 (2019).
